# The Association of Diabetes with Knee Pain Severity and Distribution in People with Knee Osteoarthritis using Data from the Osteoarthritis Initiative

**DOI:** 10.1038/s41598-020-60989-1

**Published:** 2020-03-04

**Authors:** Aqeel M. Alenazi, Mohammed M. Alshehri, Shaima Alothman, Bader A. Alqahtani, Jason Rucker, Neena Sharma, Neil A. Segal, Saad M. Bindawas, Patricia M. Kluding

**Affiliations:** 1 Department of Rehabilitation Sciences and Physical Therapy, Prince Sattam Bin Abdulaziz University, Alkharj, Saudi Arabia; 2 Department of Physical Therapy and Rehabilitation Science, University of Kansas Medical Center, Kansas City, KS USA; 3 Department of Rehabilitation Science, Jazan University, Jazan, Saudi Arabia; 4 Department of Rehabilitation Medicine, University of Kansas Medical Center, Kansas City, KS USA; 5 Department of Rehabilitation Science, King Saud University, Riyadh, Saudi Arabia

**Keywords:** Diabetes complications, Cartilage, Risk factors

## Abstract

Limited research has examined the association between diabetes mellitus (DM) and knee pain in people with osteoarthritis (OA). Therefore, this study aimed at examining the association between DM and knee pain severity, and to explore the association between DM and knee pain distribution (unilateral or bilateral versus no pain) in subjects with knee OA. This is a cross-sectional analysis of the baseline visit of individuals who were enrolled in the Osteoarthritis Initiative. Data of participants with knee OA were used for this analysis (n = 1319), and grouped into subjects with both knee OA and DM (n = 148) or knee OA only without DM (n = 1171). Pain severity was measured using a numeric rating scale from 0 to 10 over the past 7 and 30 days for each knee, and the more symptomatic knee with higher pain severity was chosen for analysis. DM was significantly associated with increased knee pain severity over 7 days (B 0.68; 95% CI 0.25–1.11) and over 30 days (B 0.59; 95% CI 0.17–1.01) after adjustments for all covariates, including age, gender, BMI, race, depression symptoms, composite OA score, use of pain medications, and knee injections. Multinomial regression showed that participants with knee OA and DM had 2.45 (95% CI 1.07–5.61) to 2.55 (95% CI 1.12–5.79) times higher likelihood of having unilateral and bilateral knee pain than those without DM and without knee pain. This study found that DM was associated with higher pain severity and unilateral and bilateral knee pain distribution.

## Introduction

Knee Osteoarthritis (OA) is the most common cause of chronic pain affecting approximately 14% of the general population^1^. Knee pain is a leading cause of disability, and the main reason for seeking medical intervention for individuals with knee OA^2^. Knee OA is currently estimated to affect approximately 37% of individuals aged ≥ 45 years, and the prevalence is expected to increase as the population of older adults continues to grow^3^. Previous research has shown that the number of comorbidities is associated with higher knee pain^4^. Among these comorbidities, metabolic syndrome, including diabetes mellitus (DM), hypertension, dyslipidemia and obesity have been related to increased pain severity among individuals with OA of knee joint^5,6^.

Diabetes is one of the most common chronic diseases, affecting approximately 10% of the general population^7^. DM is characterized by a disturbance in insulin metabolism that leads to hyperglycemia, which often leads to other complications. Hyperglycemia may induce chronic systemic inflammation that leads to systemic changes in body organs including joints^8^. Another consequence of hyperglycemia is the production of advanced glycation end products (AGE) that can accumulate in any part of the body, including the joints, and may increase cartilage stiffness and bone fragility^9^. Two recently published meta-analyses found a significant association between OA and DM^10,11^. DM may be an independent risk factor for OA progression and adverse outcomes following joint replacement^12–17^. Although knee OA progression and severity have been linked to higher body mass index^18–20^, prior research has found an association between obesity and OA in non-weight bearing joints that may suggest a systemic pathway^21,22^.

Examining associated comorbidities such as DM in people with OA is necessary to identify an increased risk of pain and multiple joint distributions, as well as to develop preventative interventions. Emerging evidence supports that patients with OA and DM have higher pain severity^12,23,24^. DM, as a systemic disease, may increase systemic inflammation that could explain higher pain severity in people with knee OA when compared to those without DM^8,23^. A recent research found a higher concentration of inflammatory markers including interleukin-6 (IL-6) in the synovial fluid and higher synovitis scores in patients with DM and end-stage knee OA^23^. However, these previous studies examined severe end-stage OA for individuals who were scheduled for arthroplasty^12,23^. Our recent work showed that increased hemoglobin A1c, a measure for average blood glucose over time, was associated with increased pain severity in patients with localized OA after controlling for using medications^25^. Previous research has mainly focused on one component of metabolic syndrome, such as obesity and its association with unilateral or bilateral knee pain, regardless of the impact of other metabolic diseases such as DM^26,27^. One common limitation in this previous research is that the effects of pain medications were not adjusted in the statistical analysis.

Understanding the association of DM with the pain experience among individuals with knee OA is valuable because it will help in designing appropriate interventions for this population. Therefore, the objectives of this study were to examine the associations of diabetes with knee pain severity and knee pain distribution (unilateral or bilateral versus no pain) in subjects with knee OA. We hypothesized that DM would be associated with a higher pain severity and more widespread distribution (e.g. bilateral knee pain) in subjects with knee OA.

## Methods

### Data source

This study is a cross-sectional analysis of the Osteoarthritis Initiative (OAI) baseline data. OAI (https://data-archive.nimh.nih.gov/oai/) is an ongoing multisite longitudinal study in the United States that enrolled 4796 participants with or at risk of knee OA to investigate the impact of knee OA over time to understand the prevention and treatment strategies better. Data were collected from four clinical centers, including Baltimore, Maryland; Columbus, Ohio; Pittsburgh, Pennsylvania; and Pawtucket, Rhode Island. Institutional Review boards for each site approved this study, and each participant signed a consent form.

### Participants

The OAI includes groups of individuals ages 45 to 79 years. This study has three cohorts: progression cohort (n = 1390 participants) who have symptomatic knee OA with both osteophytes and frequent knee symptoms in at least one knee; incidence cohort (n = 3285 participants) who have no symptomatic knee OA but are at increased risk for OA in at least one knee; and control cohort (n = 122 participants) who have no symptomatic or radiographic knee OA and no elevated risk for OA. For this study, we used baseline data only from participants in the progression cohort (n = 1390) to focus on subjects with established knee OA with radiographic evidence in at least one knee. All included participants had at least grade 2 composite OA score, equivalent to grade 2 on Kellgren and Lawrence (KL) grade in at least one knee. A self-report adaptation of the Charlson Comorbidity Index for DM (either yes or no) was used^28,29^. Past research has shown good validity ranging from 78% to 97% and reliability over time exceeding 92% of self-reported DM using self-reported questionnaires^30,31^. Participants with missing self-reported DM (n = 46) and knee joint replacement (n = 25) were excluded. Participants were further grouped into knee OA and DM (n = 148) or knee OA only without DM (n = 1171) depending on the presence or absence of DM.

### Study factors

Pain severity was measured using a numeric rating scale (NRS). Two questions were used in this study; one over seven days and the other over 30 days. The first question was: “During the past seven days, have you had this pain, aching, or stiffness in your right/left knee” if the participant answered yes, the following question was asked: “Please rate the pain that you’ve had in your right/left knee during the past seven days by pointing to the number on this card that best describes the pain at its worst. ‘0’ means ‘No pain’ and ‘10’ means ‘Pain as bad as you can imagine’”. The second question was identical except for a 30-day time frame. These questions were repeated for each knee. The more symptomatic knee was selected for the analyses in this study. If the participant answered yes to questions about pain over 30 days in both right and left knees, they were categorized as having bilateral knee pain. If they answered yes to one knee, they were categorized as having unilateral knee pain; or none if they answered no regarding both knees. Previous longitudinal studies have utilized these questions in this way^32,33^.

### Other variables

Several other variables were included in the analysis. Age, gender race, body mass index (BMI), depression symptoms, composite OA score, pain medications, and knee injections were included as covariates. Race has 4 categories including Caucasians, African American, Asians, and others. BMI was measured using body mass (kg) divided by the square of height (m) and included as a continuous covariate. Previous research showed an association between knee pain and depression in people with knee OA^34,35^. Therefore, depression was included as a covariate in the current study. Depression symptoms factor was also included as a covariate, and the participants were classified as having depression symptoms if they scored ≥16 using the Center for Epidemiologic Studies Disease (CES-D) scale^36^. Radiographic evidence of tibiofemoral knee OA at baseline, using OAI composite OA score, which can be used as a surrogate for KL grade, was included as a covariate for each participant’s knee. In addition, we included baseline KL grade for each knee as a covariate for a sensitivity analysis. Use of pain medications was included as a covariate for most commonly used pain medications for arthritis for all participants^37^. Multiple types of medications were reported via direct questions to participants and categorized separately including over the counter medications (e.g. non-prescribed non-steroidal anti-inflammatory drugs (NSAIDs) and Tylenol), prescribed non-steroidal anti-inflammatory drugs (NSAIDs), COX-2 inhibitors (coxibs), prescribed narcotics (e.g., opioids) or nutraceutical medications (e.g., S-adenosylmethionine), and having knee injection in the past 6 months. Each medication was categorized as yes if the participant reported using that medication for joint pain or arthritis more than half the days of the month during the past 30 days. This allows for controlling multiple medications for the same participant. Finally, another category was included as a covariate if the participant reported taking any pain medication on the day of the clinic visit.

### Statistical analysis

Descriptive statistics were calculated with the means for continuous variables and frequencies (percentage) for categorical variables. To compare demographics in subjects with knee OA and DM to those without DM, we used chi-square test for categorical variables and independent t-test for continuous variables. All analyses were performed using SPSS for Macintosh, version 25.0 (SPSS Inc, Chicago, IL). The significance level was set at alpha of 0.05.

To examine the association between DM and knee pain severity over 7 days and over 30 days, multiple linear regression was used. Two models were created with DM as a dummy coded factor (yes or no) and knee pain severity over 7 days and over 30 days as the outcome variables. These models included model 1 (adjusted for age, gender and BMI) and model 2 (adjusted as in model 1 in addition to race, depression symptoms, composite OA score for both knees, use of pain medications, and knee injections).

Multinomial logistic regression analysis was utilized to determine the relationship between DM and knee pain distribution. Knee pain distribution included three categories: no pain, unilateral and bilateral knee pain. Two models were created with DM as a dummy coded factor (yes or no) and joint distribution (bilateral or unilateral versus no pain) as the outcome variable. The reference category for the outcome variable was set as no pain. These models included model 1 (adjusted for age, gender and BMI) and model 2 (adjusted as in model 1 in addition to race, depression symptoms, composite OA score for both knees, use of pain medications, and knee injections). Odds ratios (OR) with associated 95% confidence intervals were calculated for each model.

### Ethical approval

All procedures performed in studies involving human participants were in accordance with the ethical standards of the institutional and/or national research committee and with the 1964 Helsinki declaration and its later amendments or comparable ethical standards. This study was approved by Institutional Review Board (IRB) for the University of California, San Francisco (UCSF) and its affiliates (approval number: FWA00000068). The IRB approval was also obtained from all the four clinical sites located at Brown University in Rhode Island, Ohio State University in Columbus, Ohio, University of Maryland/Johns Hopkins University joint center in Baltimore, Maryland, and at the University of Pittsburgh in Pennsylvania.

### Informed consent

Informed consent was obtained from all individual participants included in the study.

## Results

### Participants characteristics

Data from a total of 1319 participants were included in the analysis, due to missing data for some participants. In this sample, 1171 had knee OA without DM, and 148 had knee OA with DM. Table 1 shows the participants’ demographics and characteristics. Age and gender were not statistically different between groups (mean difference for age = 0.46 year). BMI was significantly higher in knee OA and DM group compared to knee OA only group (mean difference for BMI = 2.8 kg/m^2^). Race distribution was statistically different between groups and race categories. Knee pain over 7 days and over 30 days were significantly higher in subjects with knee OA and DM (NRS 6.07 ± 2.40 vs. 4.95 ± 2.52 for knee pain over 7 days; 6.35 ± 2.36 vs. 5.31 ± 2.45 for knee pain over 30 days) compared to subjects with knee OA only. Bilateral knee pain was present in approximately 50% of subjects with knee OA and DM and 40% of subjects with knee OA only, and this difference was statistically significant.

**Table 1:** Participants’ characteristics.

	All sample (n* = *1319)	Knee OA only (n* = *1171)	Knee OA and DM (n* = *148)	p-value
Age, years (mean ± SD)	61.20 ± 9.04	61.16 ± 9.11	61.62 ± 8.53	0.56
Female, n (%)	747 (56.6)	663 (56.6)	84 (56.8)	0.52
Race, n (%)				<0.001
Caucasians	939 (71.2)	876 (74.8)	63 (42.6)	
African American	340 (25.8)	263 (22.5)	77 (52.0)	
Asian	11 (0.8)	7 (0.6)	4 (2.7)	
Others	29 (2.2)	25 (2.1)	4 (2.7)	
BMI, kg/m^2^ (mean ± SD)	30.12 ± 4.9	29.81 ± 4.8	32.6 ± 4.9	<0.001
Knee pain over 7 days	5.08 ± 2.53	4.95 ± 2.52	6.07 ± 2.40	<0.001
Knee pain over 30 days	5.43 ± 2.46	5.31 ± 2.45	6.35 ± 2.36	<0.001
Depression symptoms, yes, n (%)	176 (13.5)	141 (12.1)	35 (24.3)	<0.001
Pain medications				
Non-prescribed NSAIDS Yes, n (%)	335 (25.4)	289 (24.7)	46 (31.1)	0.11
Tylenol, yes, n (%)	195 (14.8)	159 (13.6)	36 (24.3)	0.001
Prescribed NSAIDS, yes, n (%)	113 (8.6)	101 (8.6)	12 (8.1)	0.48
Prescribed COXIBS, yes, n (%)	139 (10.6)	131 (11.2)	8 (5.4)	0.017
Prescribed narcotics, yes, n (%)	47 (3.6)	39 (3.3)	8 (5.4)	0.15
SAMe, yes, n (%)	10 (0.8)	9 (0.8)	1 (0.7)	0.69
Any pain medication today, yes, n (%)	193 (14.6)	170 (14.5)	23 (15.5)	0.41
Knee pain distribution				
No pain, yes, n (%)	160 (12.2)	153 (13.1)	7 (4.8)	0.002
Unilateral knee pain, yes, n (%)	603 (45.9)	537 (46.0)	66 (44.9)	
Bilateral knee pain, yes, n (%)	551 (42.0)	477 (40.9)	74 (50.3)	

The p-value was obtained from chi-square test for categorical variables or independent t-test for continuous variables.

BMI: body mass index.

NSAIDS: Non-steroidal anti-inflammatory drugs.

COXIBS: cox-2 inhibitors (e.g., Bextra, Celebrex).

SAMe: S-adenosylmethionine.

### Diabetes and knee pain severity

The results of the multivariable linear regression analysis examining the association of DM with knee pain severity over 7 and 30 days are presented in Table 2 with associated 95% confidence interval (CI). Model 2 shows that DM was significantly associated with increased knee pain severity over 7 days **(**B 0.68; 95% CI 0.25–1.11) and over 30 days (B 0.59; 95% CI 0.17–1.01) after adjustments for age, gender, race, BMI, depression symptoms, composite OA score, pain medications, and knee injections.

**Table 2:** Multiple linear regression for the association between DM and knee pain severity.

Dependent variables		n	R^2^	B	SE	95% CI	*p*-value
Knee pain severity over 7 days	Model 1	1314	0.05	0.96	0.22	0.53–1.39	<0.001
	Model 2	1289	0.13	0.68	0.22	0.25–1.11	0.002
Knee pain severity over 30 days	Model 1	1316	0.06	0.88	0.21	0.45–1.29	<0.001
	Model 2	1291	0.13	0.59	0.21	0.17–1.01	0.006

n = number of patients; SE = standard error; CI = confidence interval.

The most symptomatic knee was selected for the knee pain severity.

Model 1 = adjusted for age, gender and BMI.

Model 2 = adjusted for model 1 and race, depression symptoms, composite OA score for both knees, taking pain medications, and knee injection.

### Diabetes and knee pain distribution

The results of the multinomial logistic regression analyses to examine the association between DM and joint distribution are presented in Table 3 as well as the odds ratio (OR) with associated 95% confidence interval (CI). Model 2 showed that participants with DM and knee OA had 2.45 to 2.55 times higher likelihood of having unilateral and bilateral knee pain than those without DM (OR for unilateral knee pain 2.45; 95% CI 1.07–5.61 and OR for bilateral knee pain 2.55; 95% CI 1.12–5.79) when compared to no pain in the last 30 days in either knee in subjects with frequent knee pain in the last year, after adjustments for age, gender, race, BMI, depression symptoms, composite OA score, pain medications, and knee injections.

**Table 3:** Multinomial regression for the association between DM and knee pain distribution.

Knee pain distribution		n	OR	95% CI	*p*-value
No pain			Reference	—	—
Unilateral knee pain	Model1	1311	2.51	1.12–5.63	0.024
	Model 2	1297	2.45	1.07–5.61	0.034
Bilateral knee pain	Model 1	1311	2.99	1.34–6.69	0.008
	Model 2	1297	2.55	1.12–5.79	0.026

n = number of patients; OR = odds ratio; CI = confidence interval.

Model 1 = adjusted for age, gender and BMI.

Model 2 = adjusted for model 1 and race, depression symptoms, composite OA grade, taking pain medications, and knee injection.

## Discussion

This study examined the association of DM with knee pain severity and joint distribution in individuals with knee OA. The results showed that DM was associated with higher pain severity and unilateral and bilateral joint distribution even after controlling for age gender, BMI, race, depression symptoms, composite OA score, pain medications, and knee injections.

Knee pain severity was higher in participants with DM and knee OA when compared to those with knee OA only. A few studies have examined the influence of DM on pain severity in individuals with OA and reported a negative impact of DM on knee pain^5,23,38^. These findings were consistent with our study results. Furthermore, our study explicitly examined both the short-term pain severity over 7 days and over 30 days, respectively, and DM had a negative influence on both. DM may facilitate low-grade systemic inflammation that could explain higher pain severity in people with knee OA who also have DM^8,23^. A recent study found a higher concentration of inflammatory markers including interleukin-6 (IL-6) in the synovial fluid and higher synovitis scores in patients with DM and end-stage knee OA^23^. Another study showed similar results among patients with DM who underwent knee or hip arthroplasty^12^. However, as these previous studies were conducted on subjects with advanced OA (i.e. scheduled for joint arthroplasty), their generalizability may be limited.

A common limitation in previous studies is the lack of control for pain medication usage that could affect pain severity. Pain medications introduce inter-subject variability, depending on the condition and pain severity, as well as whether they are prescription-strength or over-the-counter medications. Prescribed analgesics, in particular, could significantly affect pain severity (e.g. opioids and prescription NSAIDs). A previous report has shown that the frequency of pain medication usage was associated with increased pain severity^37^. Because using pain medication could be associated with increased pain severity, our study controlled for pain medication usage. This allows this study to have a better estimate of the influence of DM on pain severity. The results of the current study were independent of pain medication use, and DM remained significantly associated with short-term increased pain severity in subjects with knee OA.

The associations between DM and pain severity over both seven and 30 days might be clinically important with regards to the short-term association. Previous research has determined the cutoff score for minimal clinically important difference between 1 and 2 score of pain numeric rating scale^39^. The current study showed that the mean between-group differences in knee pain severity were greater than 1 point^39^. However, the adjusted linear analyses showed that participants with knee OA and DM had pain ratings over seven and 30 days respectively that were 0.68 and 0.59 points greater than those of subjects with knee OA without DM. These scores do not meet the criteria for minimal clinically important differences, suggesting that other covariates may contribute to the association between DM and pain or DM may have a weak association with pain.

Bilateral and unilateral knee pain were associated with DM in this study even after controlling for BMI, race, depression symptoms, OA grade, pain medications, and knee injections. This study found that subjects with knee OA and DM are about 2.5 times more likely to have bilateral or unilateral knee pain than subjects with knee OA without DM. These findings were different than our hypothesis that participants with DM would be more likely to have bilateral knee pain than those without DM. DM, as a systemic disease, could affect both knees in subjects with knee OA. However, since both unilateral and bilateral joint pain were significantly associated with DM, it could be that DM contributes to pain in knees that are otherwise compromised, rather than causing symptomatic knee OA. These findings are essential in considering prevention strategies for knee pain in patients with DM who are at elevated risk for knee OA. As DM appears related to bilateral and unilateral knee pain cross-sectionally, future research should advance understanding of this relationship by investigating the impact of DM and its management on worsening of knee pain in people with knee OA.

Limited research has investigated the association between metabolic disorders and knee pain distribution (e.g. bilateral knee pain). Previous work has mainly focused on one component of metabolic disorders (e.g. obesity) with conflicting results^26,27^. The current study found that another metabolic disorder, DM, was associated with unilateral and bilateral knee pain, compared to no knee pain in subjects with knee OA, independent of BMI. Prior research has mainly focused on pain severity without considering joint distribution (unilateral or bilateral) that might influence results^40,41^. People with bilateral knee pain could have more difficulty performing activities of daily living and functional activities such as climbing stairs and walking than those with unilateral knee pain^42,43^. We suspected that DM, as a systemic disease, would result in a widespread pain distribution, and be more strongly associated with bilateral versus unilateral knee pain. However, our findings indicated that DM was associated with both unilateral and bilateral knee pain. These results could be explained by recent research showing that DM was associated with accelerated cartilage degeneration^44,45^ that might affect one or both knees.

Bilateral knee pain could be an indication for OA in both knees, and it might show the associated factors with bilateral pain or multisite OA. Our recent work has shown that metabolic syndrome including DM was prevalent and associated with multisite OA or generalized OA when compared with those without DM^46^. However, previous research focusing on the association between metabolic syndrome and unilateral and bilateral knee pain has been limited to obesity as a metabolic syndrome not DM. Previous studies have found that a bilateral distribution of knee distribution was associated with higher BMI in women with knee OA^26,47^. In contrast, Frilander *et al*.^27^ did not find an association between obesity and bilateral knee pain among men. However, these reports did not examine any potential associations with DM.

Diagnosing DM is a common challenge in observational studies with large sample due the cost and lack of supply, but self-reported DM is a practical approach. The current study used self-reported DM for classifying subjects into DM group. Previous research has examined the validity and reliability for self-reported DM against medication inventory and/or glucose criteria^30,31^. Schneider *et al*. showed that both prevalent and incident DM were 84% to 97% specific, 55% to 80% sensitive, and 92% reliable over time when compared to the medication inventory and/or glucose criteria^28^. Another study by Margolis *et al*. found that self-reported DM was concordant with medication inventory in 77% of observational studies^27^. These study concluded that using self-reported DM has a sufficient accuracy and acceptable to be used in observational studies. However, there are still error estimate for self-reported DM and should considered for results interpretation.

Among the strengths of this study are adequate control for BMI as a continuous variable and the use of pain medications. The conflicting results of prior studies have examining the relationship between metabolic syndrome or diabetes and OA could be explained by inadequate controlling for BMI. In addition, this study measured pain severity in both 7- and 30-days’ time frames, extending prior research findings for the association of DM with knee pain in individuals with knee OA.

While this study has several areas of strength, some limitations should also be considered. This is a cross-sectional analysis, and the causal relationship between DM and knee pain cannot be drawn. DM was obtained by self-report, and this is a key variable in this study. There is a chance of inaccurate answers by the patients due to the presence of undiagnosed DM, denial, or lack of awareness. The results might be affected by underestimation of DM as it was a self-reported variable. DM was not specified as type1 or type 2 in this study, so both the type and duration, which may affect the results, remained uncategorized. Other DM complications such as neuropathy, ulcers, and arterial disease were not captured in this study. Glycemic control (i.e., HbA1c) was not available in the OAI and should be acknowledged as a limitation for studies with DM. Future research should investigate this association with an objectively confirmed diagnosis of DM. Although the current study found the association between DM and unilateral and bilateral knee pain, the results should be interpreted with caution. The progression cohort included participants with frequent knee pain in both groups, and this will limit the generalizability of the results to those with frequent knee pain. Although our sensitivity analysis results were similar when using OA composite score and KL grade, the results may differ when using central reading to define eligible sample. In our study, the included subjects had symptomatic knee OA defined by having grade 2 or greater using OA composite score based on clinic reading and frequent knee pain in the same knee. Therefore, the results should be interpreted with caution. Finally, other confounders were not considered such as the duration of DM and previous knee injury or surgeries. Thus, we believe that the current study findings are generalizable to broader subjects with knee OA with different stages or grades.

In conclusion, DM was associated with higher short-term pain severity when compared to subjects with knee OA only. DM was strongly associated with bilateral and unilateral knee pain relative to no knee pain as measured by self-reported knee pain over 7 and 30 days. In this cohort, subjects with knee OA and DM had a three-fold greater risk for bilateral and unilateral knee pain when compared to no pain in the last 30 days in either knee in subjects with frequent knee pain in the last year. The design of the current study prevents to determine the causal relationship between DM and knee pain in this population.

## Data Availability

The dataset generated during the current study are publicly available and can be obtained through OAI (https://data-archive.nimh.nih.gov/oai/).
